# Pachyonychia Congenita-Associated Alopecia

**DOI:** 10.1155/2012/850658

**Published:** 2012-09-29

**Authors:** Azita Nikoo

**Affiliations:** Department of Dermatopathology, Tehran University of Medical Sciences, Tehran, Iran

## Abstract

A 5-year-old female, known case of pachyonychia congenita, presented with diffuse hair loss; remaining hairs were easily plucked kinky hairs. Hair samples from patient were investigated using a light microscope. The hairs of the patients were mainly anagen hairs and unlike normal plucked anagen hairs, showed keratinization and cornification of their hair bulbs. No specific hair shaft abnormality was found.

## 1. Introduction

Pachyonychia congenita is a rare heritable disease that affects the nails, skin, oral and laryngeal mucosae, teeth, and hair [1]. Dominant-negative mutations in four keratin genes (K6a, K6b, K16, and K17) lead to keratinocyte fragility and the resultant pachyonychia congenita phenotype [2, 3]. Pachyonychia congenita is characterized by hypertrophic nail dystrophy and associated ectodermal features with subdivisions that have been suggested based on the clinical features [4].

## 2. Case Report

**Figure 1:**
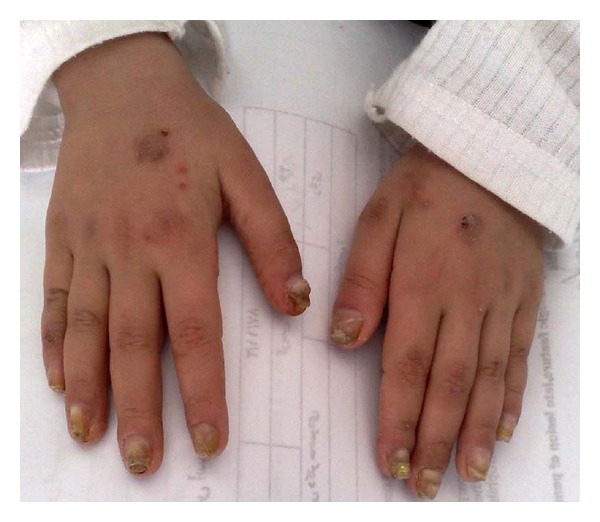
Wedge-shaped thickening, subungual hyperkeratosis, and discoloration of finger nails.

**Figure 2:**
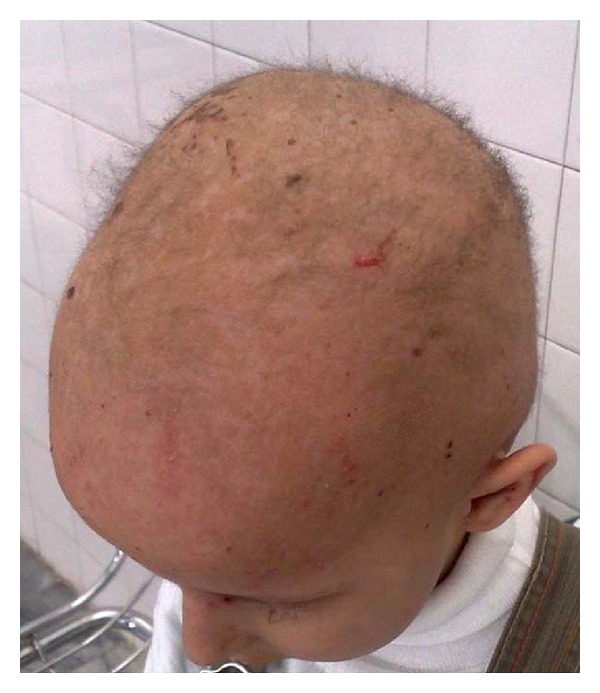
Angular cheilitis.

**Figure 3:**
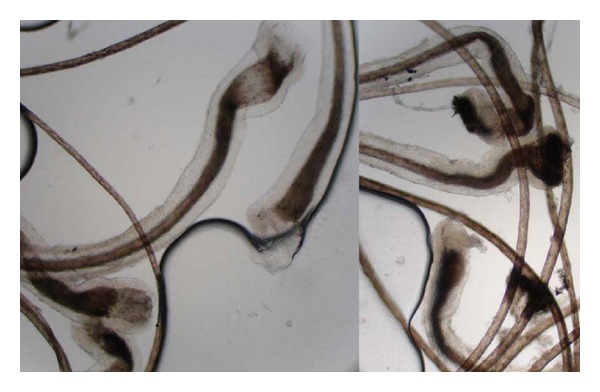
keratinization and cornification of hair bulbs. (Image of a normal terminal hair is shown for comparison.)

A 5-year-old female was admitted to our hospital with severe progressive wedge-shaped thickening, subungual hyperkeratosis, and discoloration of nails (Figure 1). These changes, which affected all 20 nails, had developed during the first year of life. She had past history of recurrent acral blisters after a few month of birth. These processes ceased after 2 years and followed by plantar keratoderma and diffuse follicular hyperkeratosis on the back and extensor areas of the limbs. There were crusted ulcers on the body and plantar areas. Further examinations showed patches of white, pseudomembranes on the buccal mucosa, and angular cheilitis. Her healthy parents had closed familial relationship. The patient's clinical presentation and history were compatible with a diagnosis of pachyonychia congenita. She also had diffuse hair loss; remaining hairs were easily plucked kinky hairs (Figure 2). Hair samples from patient were investigated using a light microscope. The hairs were mainly anagen hairs. The entire anagen hairs were cornified and showed keratinization and cornification of their hair bulbs. No specific hair shaft abnormality was found (Figure 3).

## 3. Discussion

There are a few studies about the abnormality of hairs in the pachyonychia congenita. In 2000, Selvaag and associates investigated hair samples from patients with different ectodermal dysplasias, including pachyonychia congenita by a scanning electron microscope. The hairs of the patients showed different structural abnormalities; twisted hairs, longitudinal grooves, trichorrhexis nodosa, and variations in the hair caliber, but structural hair defects were not reported in pachyonychia congenita [5]. Hair shaft abnormalities, in our patient, were not specific or significant. 

In 1997, Templeton reported the first microscopic description of a patient with pachyonychia congenita-associated alopecia (PC-associated alopecia). They suggested that the histologic presentation of PC-associated alopecia such as diminished follicular density with preservation of follicular units, prominent miniaturization of follicles, dyskeratosis of outer root sheath keratinocytes, and moderate parakeratotic and orthokeratotic follicular hyperkeratosis is very similar to that of chronic persistent or regrowth phase alopecia areata [6]. 

Anagen hairs of our patient were characterized by pigmented bulbs but like telogen hairs had cornified bulbs. Histological findings of alopecia areata such as “Exclamation mark” or tapered “pencil point” hair [7] were not found. 

We described the trichogram of a patient with PC-associated alopecia, which to our best knowledge, is reported in few articles.

## References

[1] Franklin J (1939). Pachyonychia congenita (Jadassohn and Lewandowski). *Proceedings of the Royal Society of Medicine*.

[2] Gu LH, Coulombe PA (2008). Hedgehog signaling, keratin 6 induction, and sebaceous gland morphogenesis: implications for pachyonychia congenita and related conditions. *American Journal of Pathology*.

[3] Smith FJD, Corden LD, Rugg EL (1997). Missense mutations in keratin 17 cause either pachyonychia congenita type 2 or a phenotype resembling steatocystoma multiplex. *Journal of Investigative Dermatology*.

[4] Leachman SA, Kaspar RL, Fleckman P (2005). Clinical and pathological features of pachyonychia congenita. *The Journal of Investigative Dermatology*.

[5] Selvaag E, Maseng Aas A, Heide S (2000). Structural hair shaft abnormalities in hypomelanosis of Ito and other ectodermal dysplasias. *Acta Paediatrica*.

[6] Templeton SF, Wiegand SE (1997). Pachyonychia congenita-associated alopecia. A microscopic analysis using transverse section technique. *American Journal of Dermatopathology*.

[7] Sperling LC (2003). *An Atlas of Hair Pathology with Clinical Correlations*.

